# Identification and risk stratification of coronary disease by artificial intelligence-enabled ECG

**DOI:** 10.1016/j.eclinm.2023.102259

**Published:** 2023-10-24

**Authors:** Samir Awasthi, Nikhil Sachdeva, Yash Gupta, Ausath G. Anto, Shahir Asfahan, Ruben Abbou, Sairam Bade, Sanyam Sood, Lars Hegstrom, Nirupama Vellanki, Heather M. Alger, Melwin Babu, Jose R. Medina-Inojosa, Robert B. McCully, Amir Lerman, Mark Stampehl, Rakesh Barve, Zachi I. Attia, Paul A. Friedman, Venky Soundararajan, Francisco Lopez-Jimenez

**Affiliations:** a Anumana, Inc, One Main Street, Cambridge, MA, USA; b nference, Inc, One Main Street, Cambridge, MA, USA; c Beth Israel Deaconess Medical Center, Boston, MA, USA; d Mayo Clinic, Rochester, MN, USA; e Novartis Pharmaceuticals Corporation, East Hanover, NJ, USA

**Keywords:** Artificial intelligence, ECG-AI, Coronary artery disease, Atherosclerotic cardiovascular disease, Cardiovascular risk

## Abstract

**Background:**

Atherosclerotic cardiovascular disease (ASCVD) is the leading cause of death worldwide, driven primarily by coronary artery disease (CAD). ASCVD risk estimators such as the pooled cohort equations (PCE) facilitate risk stratification and primary prevention of ASCVD but their accuracy is still suboptimal.

**Methods:**

Using deep electronic health record data from 7,116,209 patients seen at 70+ hospitals and clinics across 5 states in the USA, we developed an artificial intelligence-based electrocardiogram analysis tool (ECG-AI) to detect CAD and assessed the additive value of ECG-AI-based ASCVD risk stratification to the PCE. We created independent ECG-AI models using separate neural networks including subjects without known history of ASCVD, to identify coronary artery calcium (CAC) score ≥300 Agatston units by computed tomography, obstructive CAD by angiography or procedural intervention, and regional left ventricular akinesis in ≥1 segment by echocardiogram, as a reflection of possible prior myocardial infarction (MI). These were used to assess the utility of ECG-AI-based ASCVD risk stratification in a retrospective observational study consisting of patients with PCE scores and no prior ASCVD. The study period covered all available digitized EHR data, with the first available ECG in 1987 and the last in February 2023.

**Findings:**

ECG-AI for identifying CAC ≥300, obstructive CAD, and regional akinesis achieved area under the receiver operating characteristic (AUROC) values of 0.88, 0.85, and 0.94, respectively. An ensembled ECG-AI identified 3, 5, and 10-year risk for acute coronary events and mortality independently and additively to PCE. Hazard ratios for acute coronary events over 3-years in patients without ASCVD that tested positive on 1, 2, or 3 versus 0 disease-specific ECG-AI models at cohort entry were 2.41 (2.14–2.71), 4.23 (3.74–4.78), and 11.75 (10.2–13.52), respectively. Similar stratification was observed in cohorts stratified by PCE or age.

**Interpretation:**

ECG-AI has potential to address unmet need for accessible risk stratification in patients in whom PCE under, over, or insufficiently estimates ASCVD risk, and in whom risk assessment over time periods shorter than 10 years is desired.

**Funding:**

Anumana.

**Research in context**

**Evidence before this study**

Atherosclerotic cardiovascular disease (ASCVD) is the leading cause of death worldwide, and coronary disease is the primary driver of those mortality rates. Artificial intelligence-enabled ECG analysis (ECG-AI) has been used successfully to discriminate conditions like elevated coronary artery calcium (CAC) scores or angiographically confirmed stenosis. However, these algorithms each capture one part of the spectrum of coronary artery disease (CAD), which ranges from coronary atherosclerosis to obstructive coronary disease to having already had a myocardial infarction (MI).

**Added value of this study**

We demonstrate that three independent and performant ECG-AI models designed to detect elevated CAC score, obstructive CAD, and regional left ventricular akinesis (as an indicator of possible prior MI) function together to identify CAD and risk-stratify patients without known ASCVD. We show that ECG-AI can identify patients that are at risk for acute coronary events due to underlying CAD over timeframes as short as 3 years, even when these patients are pre-stratified by their 10-year risk for ASCVD as derived from the pooled cohort equations (PCE).

**Implications of all the available evidence**

The ECG-AI tool described here, designed to detect CAD, supports and informs clinical decision making by adding a new dimension of readily obtainable point-of-care data to ASCVD risk assessments. The tool may enable providers to titrate the strength or speed of subsequent intervention accordingly.

## Introduction

Atherosclerotic cardiovascular disease (ASCVD) is the leading cause of mortality worldwide. In the United States alone, someone will have a myocardial infarction every 40 s.^1^ Evidence-based ASCVD risk estimators have been developed to facilitate the initiation of measures for the primary prevention of ASCVD, including lipid-lowering therapy. The most widely adopted of these are the pooled cohort equations (PCE), which estimate the 10-year risk of ASCVD events (death due to coronary heart disease, nonfatal myocardial infarction, as well as fatal or nonfatal ischemic stroke) based on clinical and demographic information.^2^ The PCE and other risk estimators have known limitations, including the over- or under-estimation of risk in groups with known risk modifiers that are not included in the calculators.^3^, ^4^, ^5^, ^6^ Coronary artery calcium (CAC) scoring can provide additional value by reclassifying the estimated risk of some patients when the decision about whether to initiate therapy is unclear.^3^ However, robust evidence regarding when to obtain CAC scoring is still evolving.^7^ As such, CAC scoring remains poorly covered by health insurance plans and therefore underutilized despite widespread appreciation for its clinical utility^8^^,^^9^ and potential clinical impact^10^ were it to be supported.

Approaches that leverage artificial intelligence-enabled analysis of electrocardiograms (ECG-AI) to predict when a patient has an elevated CAC score have been developed and shown to have good discriminatory capacity. The area under the receiver operating characteristics (AUROC) for published CAC ECG-AI models have ranged from 0.75 to 0.77–0.80 for discriminating a CAC score ≥100 and a CAC score ≥300–400, respectively.^11^^,^^12^ Additionally, a recently published study demonstrated separability of ECGs coming from patients with angiographically proven severe stenosis from those of patients without CAD with an AUROC of 0.87.^13^ These ECG-AI approaches offer promise in enabling widely accessible and affordable risk stratification prior to CAC scoring.

One recent study^14^ reported that a machine learning algorithm that leverages ECG parameters in conjunction with CAC score and clinical factors outperforms either modality alone (ECG parameters, CAC score, or clinical factors) at predicting risk for a major adverse cardiovascular event. The study results suggest that the ECG offers additional, independent and untapped value for cardiovascular risk assessment.

Coronary artery disease (CAD) is a spectrum that includes risk factors, non-obstructive atherosclerosis, obstructive atherosclerosis, and myocardial infarction (which may be silent). ECG-AI designed to detect only one part of this spectrum may underperform at detecting undiagnosed CAD across the spectrum. Conversely, ECG-AI designed to detect too heterogeneous of a disease state may underperform due to impairment of the AI’s ability to learn specific pathophysiological features of high importance. We hypothesized that an ensemble of ECG-AI models trained using different stages of the CAD spectrum may perform better at identifying patients at risk for acute coronary events due to underlying CAD than a single model, when applied to patients without known ASCVD. Furthermore, we hypothesized that ECG-AI designed to detect CAD would enable the prediction of acute coronary events with greater temporal resolution than a 10-year timeframe, possibly due to the detection of subclinical or microvascular disease. Together, these advancements could enable ECG-AI to bridge the current gap between PCE-based risk estimation and non-invasive, imaging-based evaluation of CAD.

## Methods

### Data source

A deidentified,^15^ privacy-preserving database consisting of digital electronic health records (EHR) from 7,116,209 patients seen at over 70 Mayo Clinic hospitals and clinics across Arizona, Florida, Iowa, Minnesota, and Wisconsin, including 3 major health systems, were leveraged for model development and retrospective analysis. Data was obtained using the nference Analytics Platform, which converts raw EHR data from health systems into an anonymized and semi-structured database. From this database, we included all patients ≥18 years old with at least one digital 10-s, 12-lead ECG on record.

### Cohort definitions

#### Coronary artery calcium

**Fig. 1:**
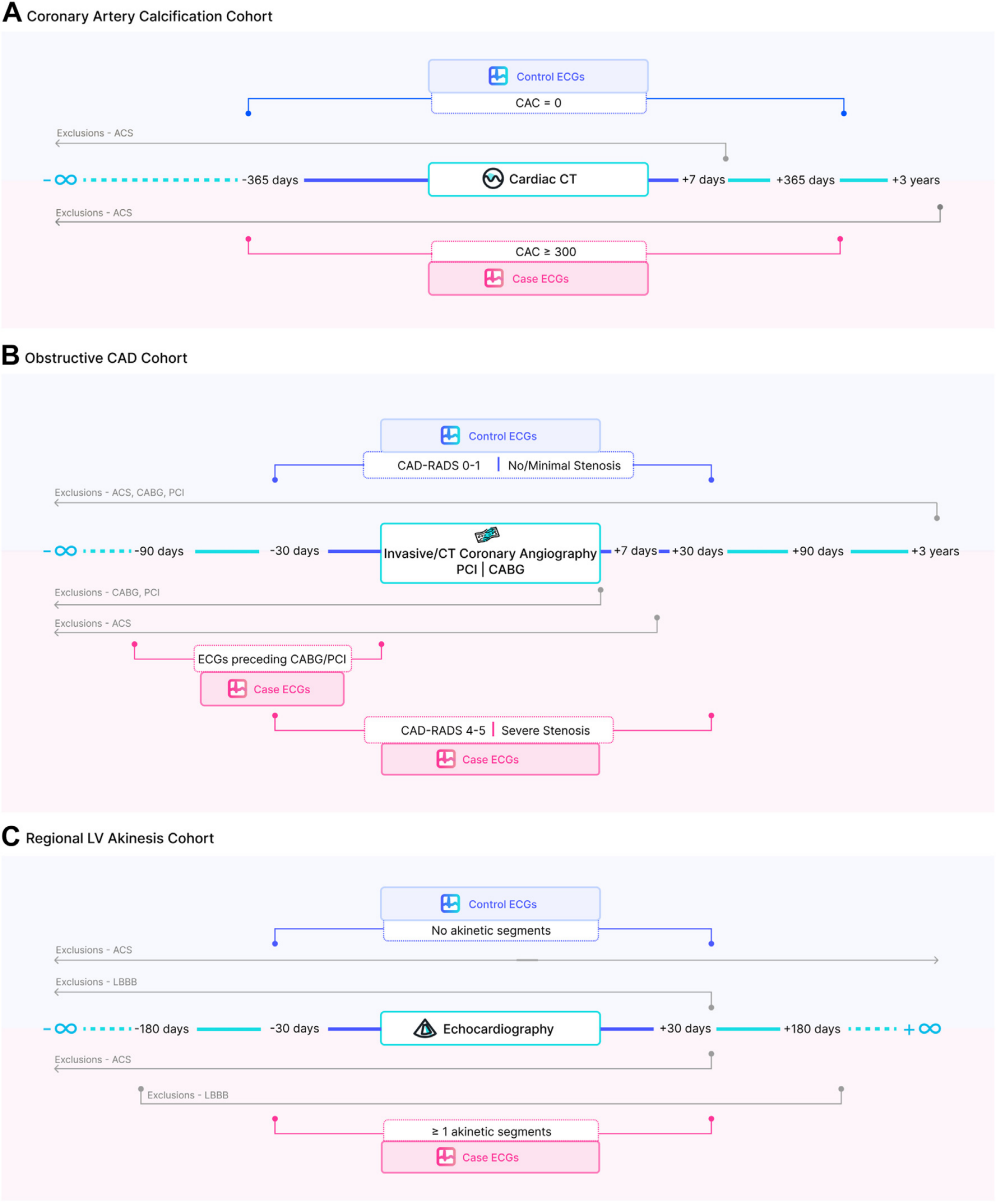
Cohort definition schematics for the three ECG-AI models developed herein, which are further detailed in the Methods section. A) Coronary artery calcium scoring from coronary CTs. B) Obstructive coronary artery diseases from coronary angiography, angioplasty and bypass grafting. C) Resting left ventricular akinesis identified via echocardiography. ACS: acute coronary syndrome; CABG: coronary artery bypass graft surgery, CAD-RADS: coronary artery disease reporting and data system, CT: computed tomography, ECG: electrocardiogram; LBBB: left bundle branch block.

CAC scores were extracted using regular expressions from the EHRs of approximately 98,684 patients that underwent an ECG-gated cardiac CT scan for CAC detection and scoring and had an ECG on record within 1 year of the CT scan, with no acute coronary syndrome (ACS) up to 7 days following the scan (Fig. 1A). A disease cohort was defined as adults with a CAC score ≥300. Controls were defined as patients who have undergone a Coronary CT with no observed coronary artery calcium (CAC score of 0 or its synonyms) and no ACS documented within the next 3 years.

#### Obstructive CAD

Disease cases were defined as adult patients that met at least one of the following criteria (Fig. 1B):

1. Underwent coronary artery bypass graft surgery (CABG) for the first time without evidence of an acute or prior ACS or prior percutaneous coronary intervention (PCI) in structured data and had an outpatient ECG within 90 days before CABG.
2. Underwent PCI for the first time without evidence of an acute or prior ACS or prior CABG in structured data and had an outpatient ECG within 90 days before PCI.
3. Underwent coronary angiography and were found to have severe stenosis and had an outpatient ECG within 30 days of angiography, with no prior PCI/CABG/ACS in structured data. Severe stenosis was defined as:
  a. ≥ 50% occlusion of left main coronary artery or
  b. ≥ 70% occlusion, including complete occlusion, of any of the other coronary arteries.
4. Underwent coronary CT angiography and were found to have severe stenosis and had an outpatient ECG within 30 days of CT angiography, with no prior PCI/CABG/ACS. Severe stenosis was defined as:
  a. Coronary Artery Disease Reporting and Data System (CAD-RADS) score of 4A, 4B or 5 in CT Coronary Angiography (CTCA).

Control cohort was defined as patients who satisfied the following criteria:

1. Underwent coronary angiography and were found to have no/minimal stenosis and had an outpatient ECG within 30 days of angiography, with no PCI/CABG before angiography or no ACS in structured data (diagnosis codes) up to 3 years after angiography. No/minimal stenosis is defined as:
  a. ≤ 25% obstruction in all coronary arteries.
2. Underwent coronary CT angiography and were found to have no/minimal stenosis and had an outpatient ECG within 30 days of CT angiography, with no PCI/CABG before CTCA or no ACS in structured data (diagnosis codes) up to 3 years after CTCA. No/minimal stenosis is defined as:
  a. CAD-RADS score of 0 or 1 on CTCA.

#### Regional left ventricular (LV) akinesis as possible prior MI

A disease cohort was defined as any adult patient with ≥1 left ventricular akinetic segment found during echocardiographic assessment of regional wall motion abnormalities (RWMA), and an ECG on record within 30 days of echocardiography (Fig. 1C). Patients are excluded from this cohort if they have diagnosis codes indicating occurrence of ACS within 180 days of echocardiography or LBBB up to 30 days after echocardiography.

A control cohort was defined as any adult patient with zero akinetic, hypokinetic, dyskinetic, or aneurysmal segments, ever, on echocardiographic RWMA assessment as well an ECG on record within 30 days of echocardiography. In addition, patients were excluded if they had a history of ACS anywhere in their lifetime, or if they had left bundle branch block (LBBB) detected up to 30 days after echocardiography.

Cohort characteristics are detailed in Table 1. Data definitions for cohort identification and curation, including the calculation of PCE scores, are detailed in the supplement. Upon implementing cohort definitions, there was natural overlap between positive cohorts (Supplementary Table S1). At the ECG level, the largest overlap was between Obstructive CAD and Regional LV Akinesis positive cohorts, with 1850 ECGs shared between cohorts (6.85% of Obstructive CAD cohort, 1.26% of Regional LV Akinesis cohort).

**Table 1:** Characteristics of development cohorts.

	Elevated CAC Score		Obstructive CAD		Regional LV Akinesis	
	Disease	Control	Disease	Control	Disease	Control
Patients						
Train	3752	6752	11,590	5888	35,220	54,123
Validate	314	529	964	478	2952	4590
Test	2190	3917	6809	3301	20,347	31,826
Total	6256	11,198	19,363	9667	58,519	90,539
ECGs						
Train	8583	8873	14,694	8288	86,357	73,844
Validate	707	687	1255	655	7246	6289
Test	5038	5221	8415	4731	50,343	43,841
Total	14,328	14,781	24,364	13,674	143,946	123,974
Demographics						
Age *[median (IQR)]*	65 (58–73)	54 (49–59)	69 (60–75)	58 (48–67)	70 (62–78)	61 (51–70)
Female	20.41%	47.2%	28.95%	52.47%	28.71%	51.16%
White	90.05%	79.68%	85.34%	87.97%	87.93%	85.95%
Black/African American	1.14%	2.73%	1.16%	3%	2.26%	4.22%
Other	0.78%	1.07%	0.93%	1.7%	1.22%	1.51%
Asian	0.41%	0.49%	0.51%	0.64%	0.46%	0.88%
Choose not to disclose	0.78%	1.84%	0.62%	1%	0.53%	0.85%
Native American/Pacific Islander	0.09%	0.2%	0.44%	0.55%	0.63%	0.5%
Unknown	6.3%	13.22%	10.44%	3.85%	6.47%	5%
Hispanic/Latino	3.06%	4.95%	1.12%	2.97%	1.82%	2.89%
Clinical characteristics at cohort entry						
Chronic IHD	55.96%	5.14%	85.13%	33.57%	79.91%	17.77%
LDL-C, mean (mg/dL) (% data availability)	104.22 (73.66%)	117.94 (60.81%)	102.48 (75.87%)	105.12 (76.42%)	92.71 (77.11%)	107.31 (78.91%)
Lp(a), mean (mg/dL) (% data availability)	39.87 (18.16%)	30.73 (17.02%)	48.61 (4.3%)	36.11 (4.37%)	49.77 (3.63%)	36.88 (4.72%)
10-year ASCVD risk (%) (% data availability)	17.63 (12.16%)	6.54 (2.64%)	23.61 (21.33%)	10.35 (26.9%)	23.74 (18.82%)	13.85 (21.12%)

ASCVD: atherosclerotic cardiovascular disease; CAC: coronary artery calcium score; CAD: coronary artery disease; IHD: ischemic heart disease; LDL-C: low density lipoprotein–calculated; LV: left ventricular; Lp(a): lipoprotein a.

##### Calculation of 10-year ASCVD risk

The pooled cohort equation (PCE) was used to compute 10-year ASCVD risk at the time of ECG. For quantitative variables, such as high density lipoprotein level, total cholesterol level, and systolic blood pressure, etc., the nearest datapoint to the ECG within 2 years prior to 1 year after an ECG was used. For diagnosis data and smoking status, both structured and unstructured data were leveraged as described above. Race/Ethnicity information was obtained directly from deidentified electronic health record (EHR). Age at the time of ECG acquisition was used. Treatment for hypertension was identified by requiring a diagnosis for hypertension and the presence of a prescription of an antihypertensive on record. The calculation of 10-year ASCVD risk using the PCE was implemented in Python.

##### Determination of occurrence of first ACS/CABG/PCI or death in EHR

For analyses in which a composite event representing acute coronary disease (ACS, CABG, or PCI) or mortality were used as outcomes, first composite event was defined as the first occurrence of a clinical diagnosis of ACS or the first occurrence of CABG/PCI in a patient’s structured and unstructured EHR using the definitions provided in Appendix A. All-cause mortality was determined from the EHR; government registries were not queried for death data because this would require the reidentification of human subjects.

### Model development

We trained separate convolutional neural networks (CNNs) to detect CAC, Obstructive CAD, and regional LV akinesis. The choice of architecture and training methods are based upon previously described successful applications of CNN architectures in the ECG-AI literature.^16^, ^17^, ^18^ Each of cohorts are split into training (60%), validation (5%), and testing (35%) datasets. To ensure data integrity, the datasets are split at the patient level, meaning each patient's ECGs are exclusively assigned to either the training, validation, or testing sets. For the training process, models are trained using the training dataset, while the validation dataset is utilized for hyperparameter tuning.

The input data expected by the algorithm consists of a 10-s signal with 12 leads, where each lead contains 5000 samples (sampled at 500Hz). Each CNN is structured with an input layer, followed by repeated blocks of convolutional layers, an adaptive pooling layer, fully connected layers, and a Softmax layer. Within each block, a Batch Normalization (BN) layer is applied before passing the data through the Rectified Linear Unit (ReLU) activation function. We used strided convolutional layers to gradually reduce the input size while simultaneously increasing the number of filters during progression through the blocks, facilitating the extraction of meaningful features. Following the final convolutional block, the output is flattened using an adaptive average pooling layer. The data then proceeds through the dense, fully connected (FC) layers. Finally, the output of the last dense layer is passed through a Softmax activation function, producing the final vector of output probabilities.

During the training process, we use the AdamW optimizer,^19^ setting the learning rate to 2e-5. Each model is initialized with pre-trained weights derived from an independently trained self-supervised learning (SSL) model; the objective of this SSL model was to learn a generic contextual representation of ECGs, and it was trained on a dataset consisting of 6 million ECGs.^20^

### Model analysis

The testing dataset, which consists of ECGs from the 35% of patients not in the training or validation datasets, was used to assess the ability of ECG-AI to detect the target condition. Because patients could contribute multiple ECGs to the dataset, a bootstrapping approach was employed to compute the receiver operating characteristic curve and subsequent performance metrics (AUROC, sensitivity, specificity). In this approach, performance metrics are repeatedly computed during 50 rounds. For each round, a single ECG is randomly selected for each patient, analyzed by ECG-AI, and compared to ground truth. Performance metrics computed from these results are averaged across rounds to obtain a patient level metric.

### Risk estimation

To understand the potential clinical utility of the ECG-AI models, we examined several cohorts of patients. We started from all patients in the EHR database with at least one digital ECG waveform available (only outpatient ECGs were used for these analyses), excluded those that were part of the training or validation sets for model development, and identified the following sub-cohorts:

1. Patients with evidence of primary care at Mayo Clinic, including all of the following:
  a. Residence in a state with Mayo primary care presence.
  b. Evidence of primary care in the EHR within 2 years of or during the observation window, including one of the following:
    i. Appointments with primary care, family medicine, or general internal medicine.
    ii. Clinical notes or ICD codes indicating a wellness or annual exam.
    iii. Evidence of primary care interventions, including vaccines on the Centers for Disease Control and Prevention adult immunization schedule or standard of care screening exams, within 2 years of or during the observation window.
  c. Patients were excluded if they had any of the following indicators of ASCVD: ACS, peripheral arterial disease, stroke, PCI, CABG, or carotid artery intervention–before cohort entry.
2. A sub-cohort of #1 aged 40–79 and with sufficient clinical data available within 2 years before to 1 year after cohort entry to calculate ASCVD risk using the PCE.^2^
3. A sub-cohort of #1 aged 18–39 with a data availability requirement identical to #2.

Event survival (acute coronary event and all-cause death) analyses were conducted on cohorts 1–3. An acute coronary event was defined as a composite occurrence of ACS, CABG or PCI. Patients that did not have a death date documented within the analysis time period or did not have follow-up until or beyond the time period were assumed to be lost to follow-up and were censored from survival analyses at the time of last encounter.

### Comparison of clinical characteristics in risk-modified patients

To understand the drivers of differences between ECG-AI-based risk classification and the calculated PCE-based risk, we compared clinical diagnoses and lab test results on record around the time of ECG-AI assessment of Cohort #2 above. For each ECG-AI model, sub-cohorts were defined:

A. Patients with low (<5%), borderline (5% ≤ risk < 7.5%), or intermediate risk by PCE (7.5% ≤ risk <20%) that are classified as CAD positive by ECG-AI
B. Patients with intermediate (7.5% ≤ risk < 20%) or high (≥20%) risk by PCE that are classified as CAD negative by ECG-AI

We then identified co-morbidities enriched in cohort A versus B in the 5 years prior to 1 month after ECG acquisition date, using structured data. We computed a rate ratio and chi square statistic to characterize the difference of co-morbidity prevalence between cohorts. Similarly, for lab results within ± 1 month with over 50% coverage of the cohorts, we computed the median and interquartile range for each lab test in each cohort and quantified the difference of the distribution of results between cohorts using Cohen’s D.^21^ For diagnoses, we rank by the chi square statistic. For lab test results, we limit to Cohen’s D ≥ 0.1.

### Statistics

**Fig. 3:**
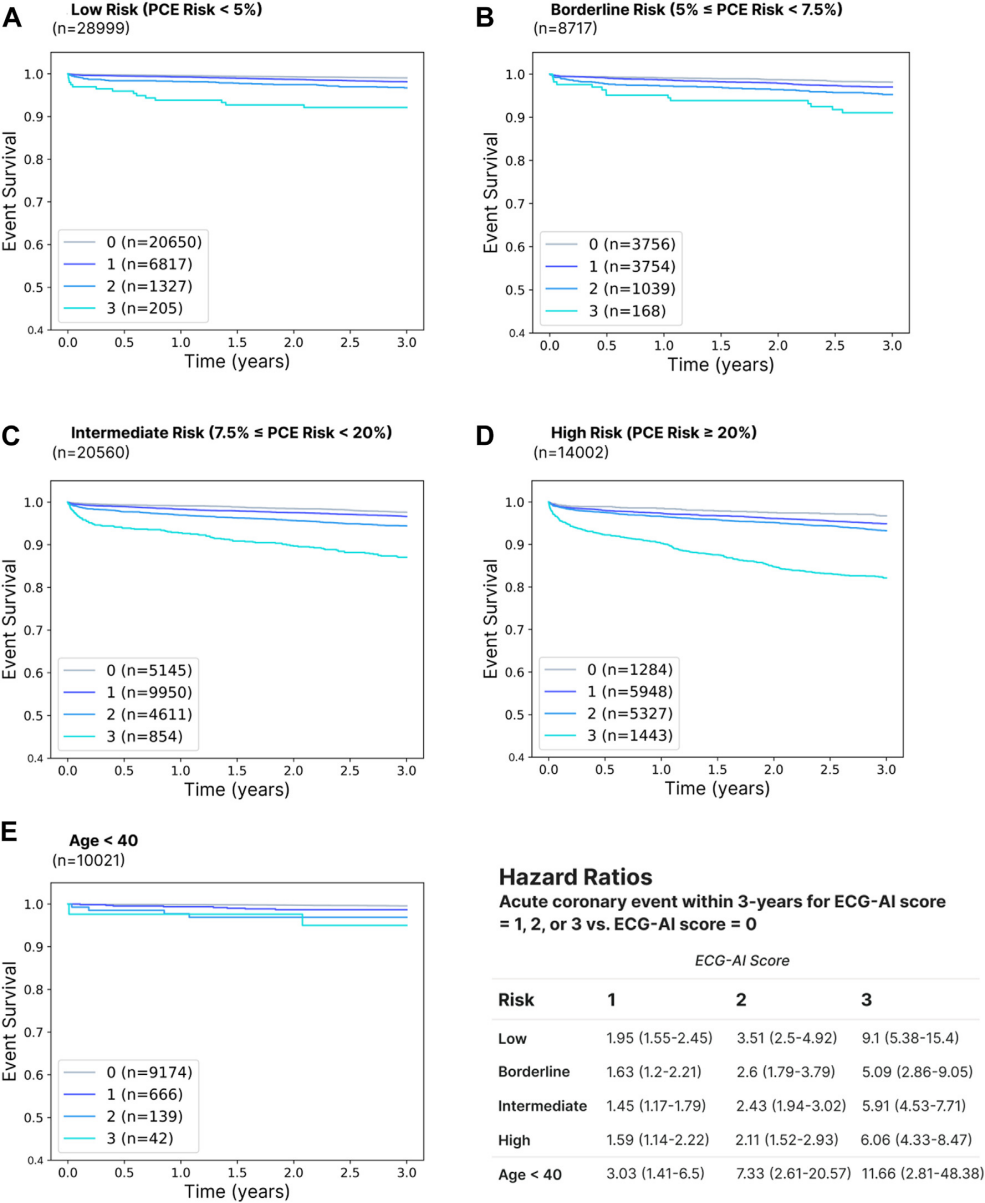
Event survival curves and hazard ratios for acute coronary events (ACS, CABG, or PCI) within a 3-year observation period in patients with no prior ASCVD (acute coronary syndrome, peripheral arterial disease, stroke, PCI, CABG, or carotid artery intervention) and low (A), borderline (B), intermediate (C), or high (D) ASCVD risk as determined by the pooled cohort equations (PCE). These results suggest an ability of ECG-AI to risk stratify patients independently of the PCE and over shorter time periods. Risk stratification is also observed in patients under the age of 40 (E). Patients were censored upon loss to follow-up if there was no EHR evidence of survival beyond the observation period or death during the observation period. For Hazard Ratios, the number in parentheses represents the 95% confidence interval. All patients were required to have evidence of primary care at Mayo Clinic. “ECG-AI score” is defined as the number of positive results on the three disease-specific models.

In general, for ECG-AI model development and analysis, patient assignment to the training, validation, or testing datasets was random. To gauge effect size differences for quantitative results, Cohen’s D^21^ was used. Cohen’s D is a metric use to describe the effect size of two distributions of data. It is computed by dividing the difference between the group means of two distributions and dividing the result by the pooled standard deviation the groups. Conventionally, a Cohen’s D of 0.2 marks a small effect size, 0.5 a medium effect size, 0.8 a large effect size, and 1.3 a very large effect size. To compare the effect of ECG-AI classification on event survival, including first acute coronary event or mortality, Cox proportional hazards modeling was used. For all analyses, sample sizes were not pre-determined; rather, all patients meeting the cohort definitions described above were studied. Cohort sizes are detailed in Table 1 and Fig. 3.

### Ethics

This research study involving deidentified EHR data of patients was conducted in strict accordance with the Health Insurance Portability and Accountability Act (HIPAA) Privacy Rule. As the study involved only deidentified data and did not involve human subjects or require identifiable information, Institutional Review Board (IRB) approval specific to this study was not required and informed consent specific to this study was not obtained. Patients that have opted out of use of EHR data for research were not included in the study. No personally identifiable information was used in the study.

### Role of the funding source

The funder was involved in the design and conduct of the study and data management. All the authors had access to the deidentified EHR records used for model development and analysis.

## Results

### Individual model performance

#### Coronary artery calcium

**Fig. 2:**
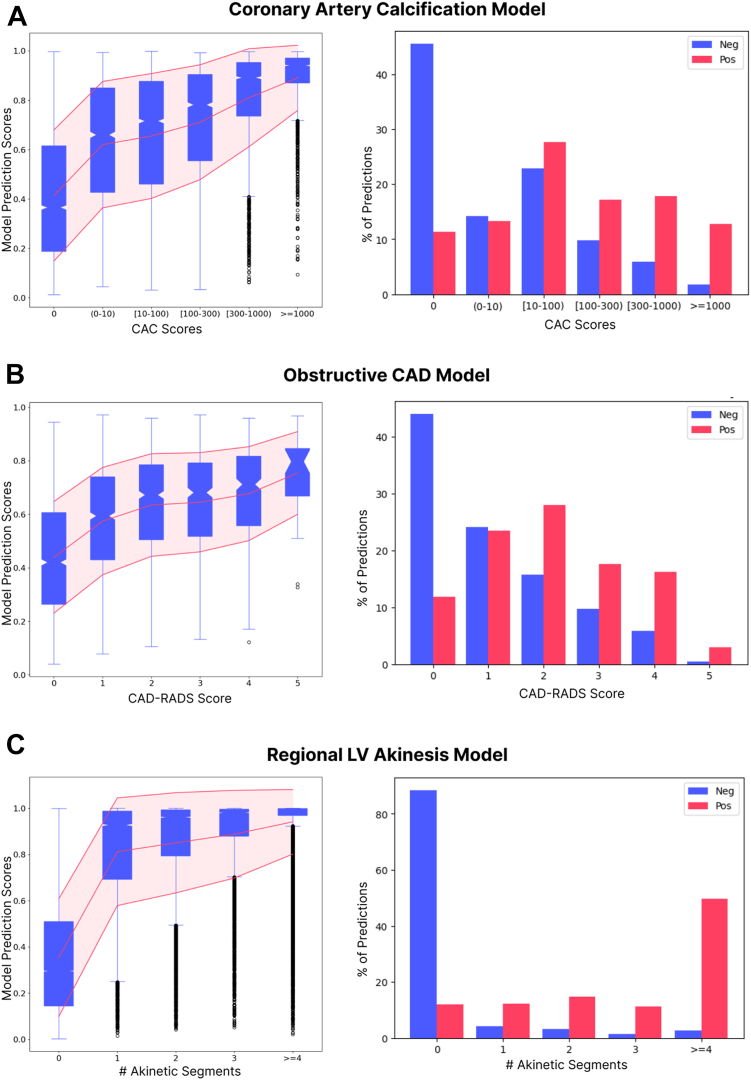
A) CAC ECG-AI model output correlates with CAC score and demonstrates an ability to discriminate elevated CAC scores. B) ObCAD model output correlates with CAD-RADS score and displays and ability to discriminate elevated CAD-RADS scores. C) Regional LV Akinesis model output correlates with number of akinetic segments and displays an ability to discriminate zero akinetic segments versus ≥1 akinetic segments. CAC: coronary artery calcium, CAD: coronary artery disease, CAD-RADS: coronary artery disease reporting and data system; LV: left ventricular. For box and whisker plots, the boxes represent the 25th through 75th percentile with the 50th percentile marked by the narrowing in between, whereas the whiskers represent the 2.5th through 97.5th percentile.

The ECG-AI model was trained to discriminate patients with CAC ≥ 300 from those with zero coronary calcification based on an ECG waveform alone and achieved an AUROC of 0.88 in a test set consisting of ECGs of previously unseen patients. At a threshold selected by optimizing the difference between the true positive rate and false positive rate (Youden’s J), the sensitivity and specificity were 78.7% and 81.6%, respectively. We also investigated the ability of the CAC ECG-AI to discriminate lower levels of atherosclerotic plaque burden. We computed the AUROC with the disease label defined as CAC ≥ 1, CAC ≥ 10, or CAC ≥ 100; the control label was kept as CAC = 0. The model achieved AUROCs of 0.79, 0.80 and 0.84 respectively. As expected, ECG-AI model scores and binary classifications were correlated with elevated calcium scores even in those patients with CACS between 0 and 300 (Fig. 2A).

#### Obstructive CAD

The ECG-AI model trained to discriminate patients with obstructive CAD from a control group achieved an AUROC of 0.85 as well as a sensitivity and specificity of 70% and 81.8% in the test dataset, respectively, at a threshold that maximizes Youden’s J. ECG-AI model scores and binary classifications were correlated with the extent of occlusion as measured by CTCA (Fig. 2B).

#### Regional left ventricular akinesis as possible prior MI

The ECG-AI model trained to discriminate patients in the test dataset with ≥1 akinetic segment from patients with no akinetic, dyskinetic, hypokinetic, or aneurysmal segments, ever, achieved an AUROC of 0.94 as well as a sensitivity and specificity of 82.2% and 92.1%, respectively, at a threshold that maximizes Youden’s J. ECG-AI model scores and binary classifications were correlated with the number of akinetic segments identified on echocardiography (Fig. 2C). Within the excluded group of patients with LBBB, the model achieves an AUROC, sensitivity, and specificity of 0.90, 0.93, and 0.63, respectively.

All results above are for patient-level classification, as described in the Methods section. AUROC curves for ECG-level classification are displayed in Supplementary Fig. S1. Sensitivity analyses in Normal Sinus Rhythm ECGs and ECGs interpreted as Normal are displayed in Table 2, and sensitivity analyses in age/sex strata are shown in Supplementary Table S2.

**Table 2:** ECG-AI model performance and comparison to conventional ECG markers of CAD.

	Coronary artery calcium	Obstructive CAD	Regional LV Akinesis
AUROC	0.88	0.85	0.94
aYouden's J threshold			
Sensitivity	0.79	0.70	0.82
Specificity	0.82	0.82	0.92
a90% Sensitivity threshold			
Sensitivity	0.93	0.90	0.90
Specificity	0.67	0.58	0.83
a90% Specificity threshold			
Sensitivity	0.74	0.57	0.85
Specificity	0.89	0.90	0.90
bConventional ECG marker performance			
Sensitivity	0.06	0.05	0.15
Specificity	0.97	0.92	0.96
Sensitivity analyses for ECG-AI			
Normal sinus rhythm ECGs (by final interpretation)			
Sensitivity	0.82	0.69	0.82
Specificity	0.81	0.85	0.93
Normal ECGs (by final interpretation)			
Sensitivity	0.74	0.66	0.37
Specificity	0.87	0.84	0.98

AUROC: area under the receiver operating characteristic; CAC: coronary artery calcium score; CAD: coronary artery disease; ECG: electrocardiogram; LV: left ventricular.

a Thresholds are calculated on validation datasets and evaluated on testing datasets.

b Conventional ECG Markers: if any one of ST depression, ST elevation, Q wave, or T wave inversion is identified in the signed ECG interpretation, the ECG is considered positive, otherwise it is considered negative.

#### Relationship between model classification and ASCVD risk factors

We examined the relationship between ECG-AI model classification and known clinical risk factors for ASCVD, including low density lipoprotein-cholesterol (LDL-C), Lipoprotein(a) (Lp(a)), and the PCE score, across a broad population by identifying all ECGs with paired risk factor data points within −2 years to +1 year of ECG acquisition for PCE score and −2 to +2 years for LCL-C and Lp(a). Only the first ECG-risk factor pair was used for analysis and patients that were members of the training or validation datasets were excluded from analysis. We used Cohen’s D to gauge the magnitude of the difference between ECG-AI positive and negative cohorts. A small effect size (Cohen’s D = −0.32) was observed for the Regional LV Akinesis model, indicating lower LDL-C in akinesis positive versus negative patients. Conversely, a small effect size (Cohen’s D = 0.19) indicated slightly higher Lp(a) in Regional LV Akinesis model positive versus negative patients. Across all models, the PCE-score was considerably higher in ECG-AI positive versus negative cohorts (Cohen’s D = 0.91, 0.96, 0.68 for CAC, Obstructive CAD, and Regional LV Akinesis ECG-AI, respectively), an effect that held upon age stratification (Supplementary Figs. S2 and S3).

### ECG-AI false positive classifications have higher cardiovascular disease burden

In order to understand what clinical features may have driven positive classifications during incorrect predictions, we compared rates of co-morbidities and medication prescriptions between the false positive and true negative classifications of each ECG-AI model.

CAC model false positives, when compared to CAC model true negatives, had higher mean age (58.6 versus 52.9) and were predominantly male (rate ratio, RR = 1.6). In the minus 1 month to +3 month time period around ECG-AI prediction, false positives were enriched for aortic valve stenosis (RR = 11.2), morbid obesity (RR = 5.2), atrial fibrillation (RR = 3.1), congestive heart failure (RR = 6.9) and essential hypertension (RR = 1.9). In the 3 months following ECG-AI prediction, false positives had higher rates of prescription for aspirin (RR = 1.6), losartan (RR = 2.8) and atorvastatin (RR = 1.3). In the 3 months following ECG-AI prediction, significantly more CT coronary angiographies (RR = 1.7), echocardiograms (RR = 1.3) and coronary angiograms (RR = 7.7) were done in false positives compared to true negatives.

Obstructive CAD model false positives, when compared to Obstructive CAD model true negatives had higher mean age (64.8 versus 55.8 years) and were predominantly male (RR = 1.3). In the minus 1 month to +3 month time period around ECG-AI prediction, false positives were enriched for atrial fibrillation (RR = 1.6), essential hypertension (RR = 1.4), aortic stenosis (RR = 2.4), congestive heart failure (RR = 1.7) and coronary atherosclerosis (RR = 1.8). In the 3 months following ECG-AI prediction, false positives had higher rates of prescription for hydralazine (RR = 2.4), clopidogrel (RR = 1.1) and warfarin (RR = 1.3). In the 3 months following ECG-AI prediction, significantly more echocardiograms (RR = 1.2) were conducted in false positives compared to true negatives.

Regional LV Akinesis model false positives, when compared to Regional LV Akinesis model true negatives had higher mean age (67.1 versus 59.6) and were predominantly male (RR = 1.3). In the minus 1 month to +3 month time period around ECG-AI prediction, false positives were enriched for coronary atherosclerosis (RR = 2.2), atrial fibrillation (RR = 3.9), congestive heart failure (RR = 7.5) and essential hypertension (RR = 1.3). In the 3 months following ECG-AI prediction, false positives had higher prescription rates for aspirin (RR = 1.6), amiodarone (RR = 15.3), metoprolol (RR = 2.1) and digoxin (RR = 6.1). In the 3 months following ECG-AI prediction, significantly more CABG (RR = 6.3), PCI (RR = 1.9) and coronary angiograms (RR = 2.1) were completed in Regional LV Akinesis model false positives compared to true negatives.

### Ensemble ECG-AI classification identifies additional risk for acute coronary events beyond the pooled cohort equations

We assessed the ability of the ECG-AI models to identify 1) acute coronary events, which are defined as the composite of ACS, CABG, or PCI; and 2) all-cause mortality. To start, we identified patients that had evidence of primary care at Mayo Clinic (Methods). Within this cohort, we examined occurrence of composite event and all-cause death over 3, 5, or 10-year observation periods following the index ECG. Patients that did not have a death date documented within the analysis time period or did not have follow-up beyond the time period were assumed to be lost to follow-up and were censored from survival analyses at the time of last encounter. In this broad cohort, which includes patients that may have substantial disease burden but no known ASCVD, we found that ECG-AI additively predicted composite event and mortality (Supplementary Fig. S4): patients that tested positive on 0, 1, 2, or 3 of the ECG-AI models had sequentially higher rates of acute coronary events or death. As expected, there was overlap between positive classifications of the different ECG-AI models. Of the Regional LV Akinesis positive ECGs, 95.6% were also positive on the CAC model and 55.3% were positive on the Obstructive CAD model (55.1% were positive on all 3 models). Of the Obstructive CAD positive ECGs, 97.7% were positive on the CAC model and 19.8% were positive on the Regional LV Akinesis model (19.7% were positive on all 3 models). Of the CAC positive ECGs, 32.4% were positive on the Obstructive CAD model and 11.3% were positive on the Regional LV Akinesis model (6.5% were positive on all 3 models).

We next asked if the risk identified by ECG-AI has clinical utility for the prediction of acute coronary events in patients without known coronary disease, beyond what is already offered by the PCE. We identified a subset of patients in whom the PCE applies: patients aged 40–79 with no prior ASCVD (ACS, PAD, stroke, CABG, PCI, or carotid artery interventions) and stratified this cohort by PCE score at cohort entry. We examined occurrence of acute coronary events over 3, 5, and 10-year observation periods, censoring patients as described above (N = 72,278, 58,799, and 25,337 patients remained uncensored for 3, 5, and 10-year periods, respectively). In the full cohort, prior to stratification by PCE score, hazard ratios for new acute coronary events in patients that tested positive on 1, 2, or 3 ECG-AI models versus 0 models were:

• 3-year: 2.41 (2.14–2.71), 4.23 (3.74–4.78), 11.75 (10.2–13.52).
• 5-year: 2.25 (2.02–2.5), 4.09 (3.66–4.56), 9.79 (8.57–11.18).
• 10-year: 2.13 (1.89–2.39), 3.67 (3.26–4.14), 8.54 (7.39–9.87).

When the cohorts were stratified by the 10-year risk for an ASCVD event as determined by the PCE, the ability to risk-stratify by ECG-AI remained strongly intact (Fig. 3A–D and Supplementary Figs. S5–S7). Similar results were observed in a cohort of patients under age 39 (Fig. 3E) and in cohorts stratified only by age (Supplementary Figs. S8).

### Comparison to standard of care evaluation for CAD

Within a cohort of patients that have evidence of primary care at Mayo Clinic, an LDL-C with blood pressure recorded within −2 to +1 years of LDL-C, and no known ASCVD prior to LDL-C, we examined the frequency of standard-of-care testing for CAD, including ECG, stress testing, and CTCA following their first qualifying LDL-C result. As expected, ECG was ordered far more frequently than either stress testing or CTCA (Supplementary Table S3). Starting from the time of cohort entry, we observed that ECGs were ordered in 36.3%, 47.7%, and 71.6% of patients over 3, 5, and 10-year observation periods, respectively. Conversely, stress testing was ordered for 9.3%, 13.4%, and 24.7% of patients and CTCA was ordered for 0.4%, 0.6%, and 1.54% of patients. When examining the subset of patients that later had an acute coronary event within the specified timeframe, patients received an ECG prior to the acute coronary event in 57.6%, 61.8%, and 74.3% of patients for the 3, 5, and 10-year observation periods, respectively. Stress testing and CTCA were obtained much less frequently prior to acute coronary event (stress test: 22.2%, 26.2%, and 34.5%, and CTCA: 2.1%, 1.8%, 2.5%). Of the patients with ECGs available prior to coronary event, over 60% tested positive on two or more of the ECG-AI algorithms described herein and over 88% tested positive on at least one. Conversely, only 13.1%, 15.0%, and 18.0% of ECGs in the 3-, 5-, and 10-year cohorts, respectively, were positive for one or more conventional ECG markers of coronary disease (T wave inversion, Q waves, ST depression, ST elevation).

### Clinical characteristics of risk-modified patients

The ability of CAD ECG-AI algorithms to stratify a patient’s risk in an additive fashion raises the question of what clinical factors are associated with ECG-AI-based risk modification. We subset patients with a paired ECG and PCE score into two groups: (A) Low or Indeterminate risk for ASCVD (<20%) but ECG-AI positive, and (B) Indeterminate or High risk for ASCVD (>7.5%) but ECG-AI negative. We compared diagnostic histories in the 5 years before to 30 days after cohort entry in cohort A versus cohort B for each of the 3 models (Table 3), as well as laboratory results in the 30 days prior to 30 days after cohort entry (Supplementary Table S4).

**Table 3:** Clinical correlates of ECG-AI classification when discordant with PCE-based ASCVD risk.

Coronary artery calcium						
Disease	Risk increase		Risk decrease		bRate ratio	aChi square
	Patient #	%	Patient #	%		
First degree atrioventricular block	1397	4.94	67	1.01	4.90	207.45
Obstructive sleep apnea	4873	17.23	677	10.18	1.69	200.57
Atrial fibrillation	2326	8.22	218	3.28	2.51	195.27
Congestive heart failure	1173	4.15	66	0.99	4.18	156.77
Hyperlipidemia	16,677	58.97	4471	67.2	0.88	152.92
morbid obesity	2451	8.67	285	4.28	2.02	143.31
Tobacco use disorder	3615	12.78	556	8.36	1.53	100.30
Mitral valve insufficiency	715	2.53	52	0.78	3.23	76.51
Chronic kidney failure	472	1.67	27	0.41	4.11	61.03
Left bundle branch block	457	1.62	28	0.42	3.84	56.18

Obstructive coronary artery disease						
Disease	Risk increase		Risk decrease		Rate ratio	Chi square
	Patient #	%	Patient #	%		
Tobacco use disorder	980	13.84	2047	8.72	1.59	159.81
Coronary atherosclerosis of native coronary artery	299	4.22	435	1.85	2.28	130.30
Type 2 diabetes mellitus	1187	16.76	5362	22.84	0.73	119.32
Colonic diverticulosis	956	13.5	4213	17.95	0.75	76.48
Postmenopausal	127	1.79	902	3.84	0.47	70.17
Hypercalcemia	105	1.48	769	3.28	0.45	62.94
Impotence of organic origin	585	8.26	1343	5.72	1.44	59.41

Regional left ventricular akinesis as possible prior myocardial infarction						
Disease	Risk increase		Risk decrease		Rate ratio	Chi square
	Patient #	%	Patient #	%		
Congestive heart failure	632	22.63	730	2.36	9.58	2711.16
Dilated cardiomyopathy	181	6.48	42	0.14	47.67	1567.82
Left bundle branch block	248	8.88	256	0.83	10.71	1126.41
Atrial fibrillation	640	22.91	2362	7.65	3.00	735.59
Tricuspid valve disease	314	11.24	697	2.26	4.98	710.47
Cardiac pacemaker in situ	115	4.12	90	0.29	14.13	619.91
Mitral valve insufficiency	247	8.84	566	1.83	4.83	534.61
Hypertrophic cardiomyopathy	76	2.72	67	0.22	12.55	379.99
Pleural effusion	224	8.02	701	2.27	3.53	317.20
Paroxysmal ventricular tachycardia	92	3.29	153	0.5	6.65	277.86

a Shown are the most significant differences (ranked by Chi square) in diagnosis code history within −5 years to +30 days cohort entry between two cohorts: Cohort A, Low or indeterminate risk for ASCVD (<20%) but ECG-AI positive; and Cohort B, Indeterminate or high risk for ASCVD (>7.5%) but ECG-AI negative.

b A rate ratio >1 indicates a higher rate in Cohort A versus Cohort B.

Notable correlates of positive risk modification by ECG-AI included atrioventricular block and obstructive sleep apnea for the CAC model; congestive heart failure, atrial fibrillation, valvular heart disease, and LBBB for both the CAC and Regional LV Akinesis models; and tobacco use disorder and impotence for obstructive CAD.

Notable correlates of negative risk modification by ECG-AI included post-menopausal state, hypercalcemia, diverticulosis, and type 2 diabetes for Obstructive CAD.

## Discussion

In this study, we developed three ECG-AI models to identify CAD, including elevated CAC score, Obstructive CAD, and Regional LV Akinesis as a possible indicator of a prior MI. The individual models had excellent performance and good correlation with known clinical and laboratory risk factors for ASCVD. We found that the three models, when ensembled into a single model, combined to provide additive information to a patient’s standard-of-care PCE-based cardiovascular risk assessment using a routine and affordable ECG test. Most strikingly, ECG-AI identified patients with elevated acute coronary event risk over timeframes as short as 3 years, even within cohorts already stratified by 10-year ASCVD risk. We hypothesize that the underlying neural networks are identifying complex patterns associated with CAD that enable the ECG-AI to detect microvascular and subclinical or silent coronary disease that may be present despite no prior ASCVD diagnosis on record.

The PCE is a foundational component of the current clinical practice guidelines for primary prevention of ASCVD.^22^ PCE is based on patient factors such as age, sex, race, blood pressure, cholesterol, diabetes and smoking history^2^ and is used in primary prevention to stratify patients based on predicted 10-year risk for ASCVD-related acute events. It informs patient-provider discussions on risk prevention in patients between 40 and 79 years of age without a history of ASCVD. Statin therapy is often recommended based on the results of the PCE and ensuing patient-provider risk discussion and shared decision making. For patients in the Intermediate Risk category, CAC testing can also help decision-making for initiating statins.^22^ PCE-based estimations of 10-year ASCVD risk were significantly higher in ECG-AI positive versus negative patients across all models (Supplementary Figs. S2 and S3) suggesting that the ECG-AI models may be used as adjuncts to existing PCE-based estimations of risk in the clinical setting. Furthermore, when examining outcomes over 3, 5 and 10 years in patients without diagnosed ASCVD, ECG-AI positive patients had higher rates of mortality and MI compared to negative patients, regardless of the model used (Supplementary Fig. S4). In cohorts stratified by a clinically applicable PCE score, in patients aged 18–39, and in cohorts stratified by age alone, acute coronary event and mortality risk increased stepwise with the number of positive ECG-AI results (Fig. 3 A–E and Supplementary Figs. S5–S8). These results suggest that ECG-AI models designed to identify CAD can provide additional, previously unavailable information to a CAD risk assessment and therefore serve as a powerful tool for MI risk prediction. The ability of ECG-AI to predict MI risk in timeframes as short as 3-years is especially compelling because it may prompt more aggressive, collaborative decision making to definitively diagnose and treat high risk ASCVD.

ECG-AI is uniquely positioned to be implemented quickly and widely due to the ubiquitous nature of the 12-lead ECG. We examined rates of clinical testing for CAD in patients that did not have known ASCVD yet went on to experience an acute coronary event within 3-, 5-, or 10-year timeframes. We found that ECGs were performed far more frequently than stress testing or CTCA. Over 88% of those patients tested positive on at least one of the ECG-AI models described herein and over 60% tested positive on at least two. Conversely, fewer than 13–18% had conventional ECG markers of CAD. These results suggest that ECG-AI offers immediate opportunity for increasing diagnosis rates of life-threatening CAD.

We also sought to identify ECG characteristics the models presented here might be using to identify CAD. While neural networks like these are inherently “black box”-like, we may gain insight into their function by examining patterns in the characteristics of ECGs associated with positive or negative ECG-AI classifications. These characteristics can be derived from automated ECG analyses, such as those performed by manufacturer ECG analysis programs. Using the 3-, 5-, and 10-year cohorts, we compared the values of 60+ lead-level parameters between ECG-AI positive and negative classifications for each of the three models. For the CAC model, ECGs classified as positive tended to have increased QRS duration, lower T-wave amplitude, reduced T-wave areas, depressed ST amplitudes, and lower QRS balance when compared to ECGs classified as negative (all effects reported had at least small to medium effect sizes, Cohen’s D ≥ 0.45). For the Obstructive CAD model, ECGs classified as positive tended to have lower R-peak amplitude, lower QRS balance, reduced T-wave area and depressed ST amplitudes when compared to ECGs classified as negative (effect sizes were smaller, with Cohen’s D ≥ 0.25). For the Regional LV Akinesis model, ECGs classified as positive tended to have longer QRS duration, lower QRS balance, lower T-wave amplitude, depressed ST amplitudes, and reduced T-wave areas (effect sizes were medium or greater, Cohen’s D ≥ 0.5). While these patterns are known to be associated with CAD, neural networks are likely able to analyze them, as well as other patterns not described here, in relation to one another in ways that the human mind cannot efficiently accomplish.

This work has several limitations. First, the data come from regions of the US that, though geographically diverse, do not fully reflect the racial and ethnic diversity of the US or global population. Further evaluation with additional datasets are needed to validate the model performance and generalizability of these novel and ensembled ECG-AI models. Additionally certain ECG-AI algorithms developed using the same dataset have demonstrated generalizability across race/ethnicity.^23^ Second, all disease and control cohorts had median ages at or above 60; patients in which primary prevention is most impactful, those under 55, are underrepresented. Third, the analyses of the ability of the three ECG-AI models to identify risk are limited by their retrospective, real-world nature. The inclusion criteria for each patient in these analyses was that they were evaluated by ECG and have one in their EHR; such an encounter represents clinical decision-making that is itself tied to risk in a patient. This risk is reflected in the high baseline mortality rates in the 3, 5, and 10-year observational cohorts relative to the general population. Nevertheless, the ECG-AI demonstrated an ability to risk stratify within this population.

In summary, these data demonstrate that ECG-AI has discriminant value at multiple points along the spectrum of coronary disease. ECG-AI developed to identify 1) elevations in CAC, 2) Obstructive CAD, and 3) Regional LV Akinesis as a marker of possible prior MI are complementary and identify unique risk profiles. ECG-AI disease classification corresponds to significantly elevated PCE-derived 10-year risk and the individual ECG-AI models combine to identify additive risk for MI or death over timeframes as short as 3 years. These data suggest that ECG-AI designed to detect CAD may be able to support and inform clinical decision making by adding another dimension of easily obtainable, point-of-care data to ASCVD risk assessments, enabling providers to titrate the strength or speed of subsequent intervention accordingly.

## Contributors

• Samir Awasthi: Conceptualization, Data curation, Formal analysis, Funding acquisition, Investigation, Methodology, Project administration, Resources, Supervision, Validation, Visualization, Writing—original draft, Writing—review & editing.

• Nikhil Sachdeva: Data curation, Formal analysis, Methodology, Software, Validation, Visualization, Writing—original draft.

• Ruben Abbou: Data curation, Formal analysis, Methodology, Software, Validation, Writing—original draft.

• Yash Gupta: Data curation, Formal analysis, Methodology, Project administration, Software, Supervision, Validation, Visualization, Writing—original draft.

• Ausath G. Anto: Data curation, Formal analysis, Investigation, Validation, Writing—original draft.

• Shahir Asfahan: Data curation, Formal analysis, Methodology, Project administration, Supervision, Validation, Writing—original draft.

• Sairam Bade: Data curation, Formal analysis, Visualization, Writing—review & editing.

• Sanyam Sood: Data curation, Formal analysis, Visualization.

• Lars Hegstrom: Data curation, Investigation, Visualization, Writing—original draft.

• Nirupama Vellanki: Validation, Writing—original draft.

• Heather M. Alger: Project administration, Writing—original draft.

• Melwin Babu: Resources, Supervision, Writing—review & editing.

• Jose R. Medina-Inojosa: Writing—review & editing.

• Robert B. McCully: Methodology, Writing—review & editing.

• Amir Lerman: Methodology, Writing—review & editing.

• Mark Stampehl: Conceptualization, Funding acquisition, Writing—review & editing.

• Rakesh Barve: Resources, Supervision, Writing—review & editing.

• Paul A. Friedman: Conceptualization, Writing—review & editing.

• Zachi I. Attia: Writing—Conceptualization, Writing—review & editing.

• Venky Soundararajan: Conceptualization, Funding acquisition, Resources, Writing—review & editing.

• Francisco Lopez-Jimenez: Conceptualization, Funding acquisition, Methodology, Supervision, Validation, Writing—review & editing.

The following authors verified the underlying data: SA, NS, RA, YG, AGA, SA, FLJ.

All authors read and approved the final version of the manuscript.

## Data sharing statement

This study involves analysis of de-identified Electronic Health Record (EHR) data via the nference Analytics Platform. Data shown and reported in this manuscript has been extracted from this environment using an established protocol for data extraction, aimed at preserving patient privacy. The data has been de-identified pursuant to an expert determination in accordance with the HIPAA Privacy Rule. Any data beyond what is reported in the manuscript, including but not limited to the raw EHR data, cannot be shared or released due the parameters of the expert determination to maintain the data de-identification. Contact corresponding authors for additional details regarding the nference Analytics Platform.

## Declaration of interests

SA, NS, RA, YG, AGA, SA, SB, SS, LH, NV, HA, MB, RB, and VS were employees of nference, inc. and/or Anumana, inc. at the time the work was conducted and held vested or unvested stock. MS is an employee and stockholder of Novartis Pharmaceuticals Corporation. PAF, ZIA, and FLJ are advisors to Anumana. In conjunction with Mayo Clinic, PAF, ZIA, FLJ, and JMRI have filed patents related to the application of AI to the ECG for diagnosis and risk stratification.
