# Supplementary material for: Identification and risk stratification of coronary disease by artificial intelligence-enabled ECG
Source: eClinicalMedicine. 2023 Oct 24;65:102259. doi: 10.1016/j.eclinm.2023.102259 (PMC10725070; doi:10.1016/j.eclinm.2023.102259)
Supplement: Supplementary Figs. S1–S8 and Tables S1–S4 [file mmc1.docx]

**SUPPLEMENTARY INFORMATION**

**Supplementary Table 1**

| **Patient-level Overlap in ECG-AI Development Cohorts** | | | | | | |
| --- | --- | --- | --- | --- | --- | --- |
|  |  |  |  |  |  |  |
|  | **Reg. LV Ak (+)** | **Reg. LV Ak (-)** | **ObCAD (+)** | **ObCAD (-)** | **CAC (+)** | **CAC (-)** |
| **Reg. LV Ak** (+)** | 58519 | 0 | 2953 | 0 | 297 | 37 |
| **Reg. LV Ak (-)** | 0 | 90539 | 773 | 1007 | 919 | 1091 |
| **ObCAD* (+)** | 2953 | 773 | 19363 | 0 | 509 | 21 |
| **ObCAD (-)** | 0 | 1007 | 0 | 9667 | 98 | 321 |
| **CAC (+)** | 297 | 919 | 509 | 98 | 6256 | 0 |
| **CAC (-)** | 37 | 1091 | 21 | 321 | 0 | 11198 |
|  |  |  |  |  |  |  |
|  |  |  |  |  |  |  |
| **ECG-level Overlap in ECG-AI Development Cohorts** | | | | | | |
|  |  |  |  |  |  |  |
|  | **Reg. LV Ak (+)** | **Reg. LV Ak (-)** | **ObCAD (+)** | **ObCAD (-)** | **CAC (+)** | **CAC (-)** |
| **Reg. LV Ak (+)** | 143946 | 0 | 1850 | 0 | 233 | 27 |
| **Reg. LV Ak (-)** | 0 | 123974 | 282 | 474 | 372 | 513 |
| **ObCAD (+)** | 1850 | 282 | 24364 | 0 | 486 | 25 |
| **ObCAD (-)** | 0 | 474 | 0 | 13674 | 91 | 353 |
| **CAC (+)** | 233 | 372 | 486 | 91 | 14328 | 0 |
| **CAC (-)** | 27 | 513 | 25 | 353 | 0 | 14781 |

** ObCAD – Obstructive CAD*

***Reg. LV Ak – Regional Left Ventricular Akinesis*

**Supplementary Figure 1**


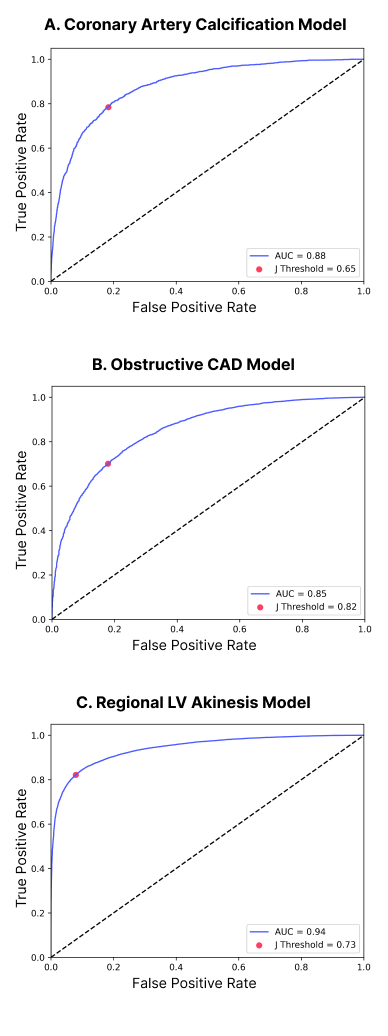


**Supplementary Figure 1.** AUROC curves with J Threshold of (A) CAC model, (B) Obstructive CAD model and (C) Regional LV akinesis model.

**Supplementary Table 2**

**ECG-AI Sensitivity Analyses: Age and Biological Sex**

|  | **CAC ECG-AI** | | | **Obstructive CAD ECG-AI** | | | **Regional LV Akinesis ECG-AI** | | |
| --- | --- | --- | --- | --- | --- | --- | --- | --- | --- |
| **Age / Sex Strata** | **AUC** | **Sensitivity** | **Specificity** | **AUC** | **Sensitivity** | **Specificity** | **AUC** | **Sensitivity** | **Specificity** |
| **ALL** | 0.882 | 0.787 | 0.816 | 0.849 | 0.7 | 0.818 | 0.941 | 0.822 | 0.921 |
| **Age 0-40 MALE** | 0.866 | 0.6 | 0.919 | 0.834 | 0.365 | 0.946 | 0.951 | 0.78 | 0.959 |
| **Age 0-40 FEMALE** | 1 | 1 | 0.984 | 0.919 | 0.36 | 0.993 | 0.955 | 0.721 | 0.978 |
| **Age 40-50 MALE** | 0.798 | 0.539 | 0.869 | 0.845 | 0.568 | 0.887 | 0.944 | 0.802 | 0.954 |
| **Age 40-50 FEMALE** | 0.774 | 0.12 | 0.948 | 0.79 | 0.342 | 0.94 | 0.94 | 0.748 | 0.974 |
| **Age 50-60 MALE** | 0.772 | 0.67 | 0.736 | 0.827 | 0.677 | 0.812 | 0.942 | 0.815 | 0.934 |
| **Age 50-60 FEMALE** | 0.816 | 0.48 | 0.957 | 0.761 | 0.427 | 0.903 | 0.926 | 0.71 | 0.968 |
| **Age 60-70 MALE** | 0.73 | 0.83 | 0.447 | 0.803 | 0.755 | 0.666 | 0.921 | 0.815 | 0.892 |
| **Age 60-70 FEMALE** | 0.793 | 0.602 | 0.864 | 0.78 | 0.524 | 0.829 | 0.923 | 0.74 | 0.952 |
| **Age 70-80 MALE** | 0.689 | 0.925 | 0.19 | 0.797 | 0.841 | 0.577 | 0.923 | 0.867 | 0.822 |
| **Age 70-80 FEMALE** | 0.764 | 0.8 | 0.59 | 0.774 | 0.628 | 0.753 | 0.904 | 0.744 | 0.927 |
| **Age 80+ MALE** | 0.534 | 0.995 | 0.042 | 0.716 | 0.85 | 0.436 | 0.918 | 0.903 | 0.731 |
| **Age 80+ FEMALE** | 0.889 | 0.975 | 0.307 | 0.728 | 0.627 | 0.737 | 0.914 | 0.84 | 0.849 |

**Supplementary Figure 2**


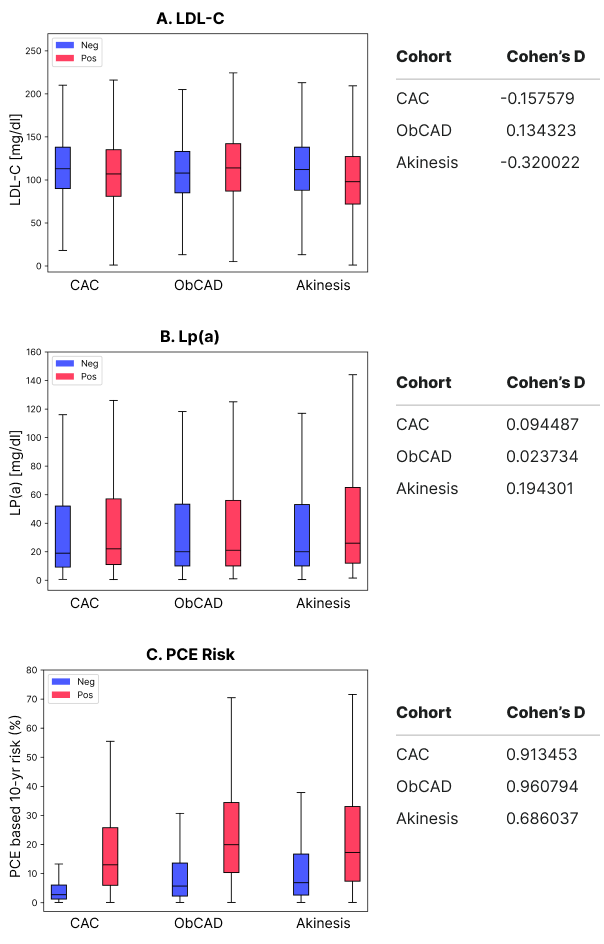


**Supplementary Figure 2.** Comparison of known clinical and demographic risk factors for ASCVD with ECG-AI classification. A) The median (IQR) LDL-C in mg/dL for each model classification was: CAC negative, 113.0 (90.0-138.0); CAC positive, 107.0 (81.0-135.0); ObCAD negative, 108.0 (85.0-133.0); ObCAD positive, 114.0 (87.0-142.0); Akinesis negative, 112.0 (88.0-138.0); Akinesis positive, 98.0 (72.0-127.0). (B) For Lp(a), the median (IQR) Lp(a) in mg/dL level for each model classification was: CAC negative, 19 (9.2-52); CAC positive, 22 (11-57); ObCAD negative, 20 (10-53.3); ObCAD positive, 21 (10-56); Akinesis negative, 20 (10-53); Akinesis positive, 26 (12-65). (C) For 10-year ASCVD risk as determined by the PCE (Figure 3C), the median (IQR) PCE for each model classification was: CAC negative, 2.7% (1.3%-6.1%); CAC positive, 13.0% (6.0%-25.8%); ObCAD negative, 5.7% (2.3%-13.6%); ObCAD positive, 19.9% (10.3%-34.4%); Akinesis negative, 6.9% (2.6%-16.7%); Akinesis positive, 17.3% (7.4%-33.0%). Bars represent the median and IQR while whiskers represent the minimum and maximum values in the dataset after excluding outliers (1.5 x IQR).

**Supplementary Figure 3**


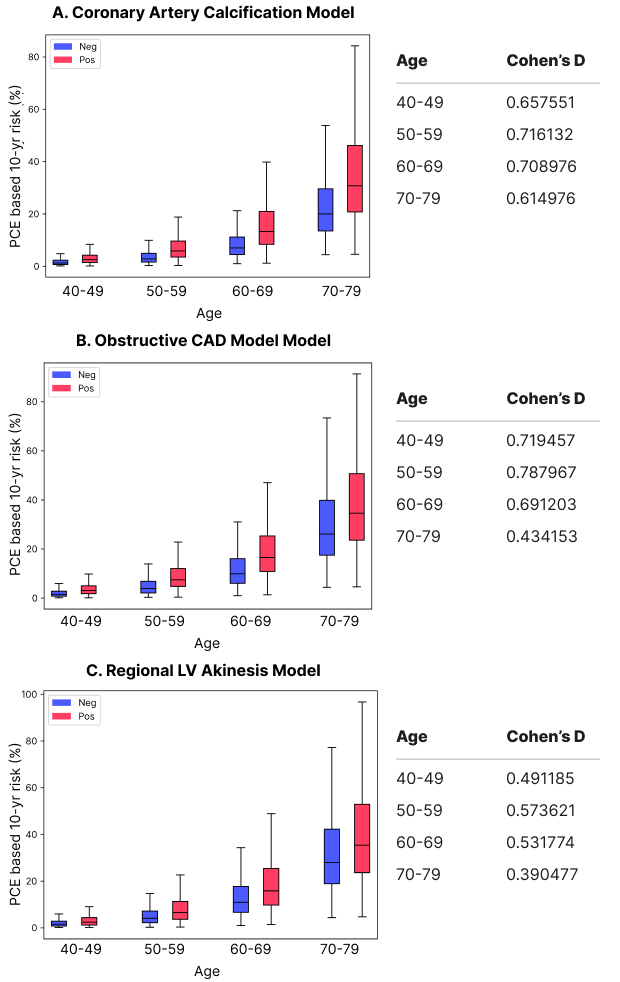


**Supplementary Figure 3.** PCE-derived 10-year ASCVD risk in ECG-AI positive versus negative cohorts stratified by age for the (A) CAC, (B) Obstructive CAD, and (C) Regional LV Akinesis models. Cohen’s D is used to determine the effect size. Notably, upon age stratification, all ECG-AI models continue to demonstrate large effect size differences in the positive versus negative classes.

**Supplementary Figure 4**


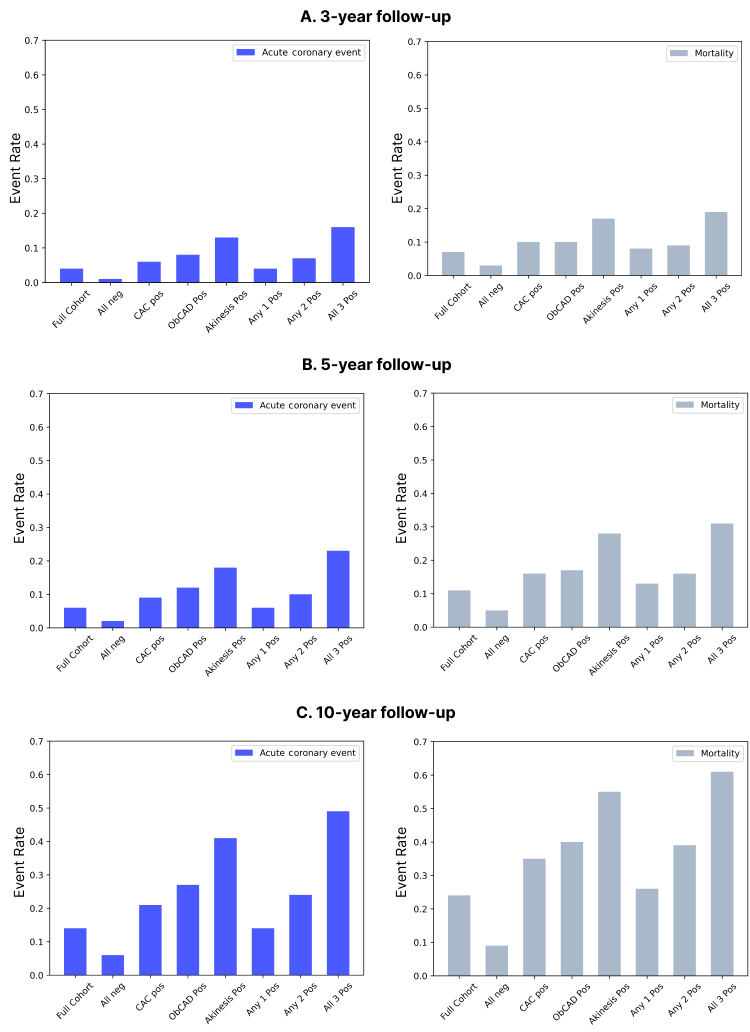


**Supplementary Figure 4.** Event rates for acute coronary events (left column) and all-cause death (right column) over (A) 3-year timeframes, (B) 5-year timeframes, and (C) 10-year timeframes for patients that test positive on 0, 1, 2, or 3 ECG-AI models. Also shown are event rates patients that test positive on specific ECG-AI models. All patients had no diagnosed ASCVD at the time of cohort entry (time of ECG-AI analysis). The cohorts used for this analysis are described in the Methods section and are the same as the cohorts used for Figures 3 and Supplementary Figures 5-8.

**Supplementary Figure 5**
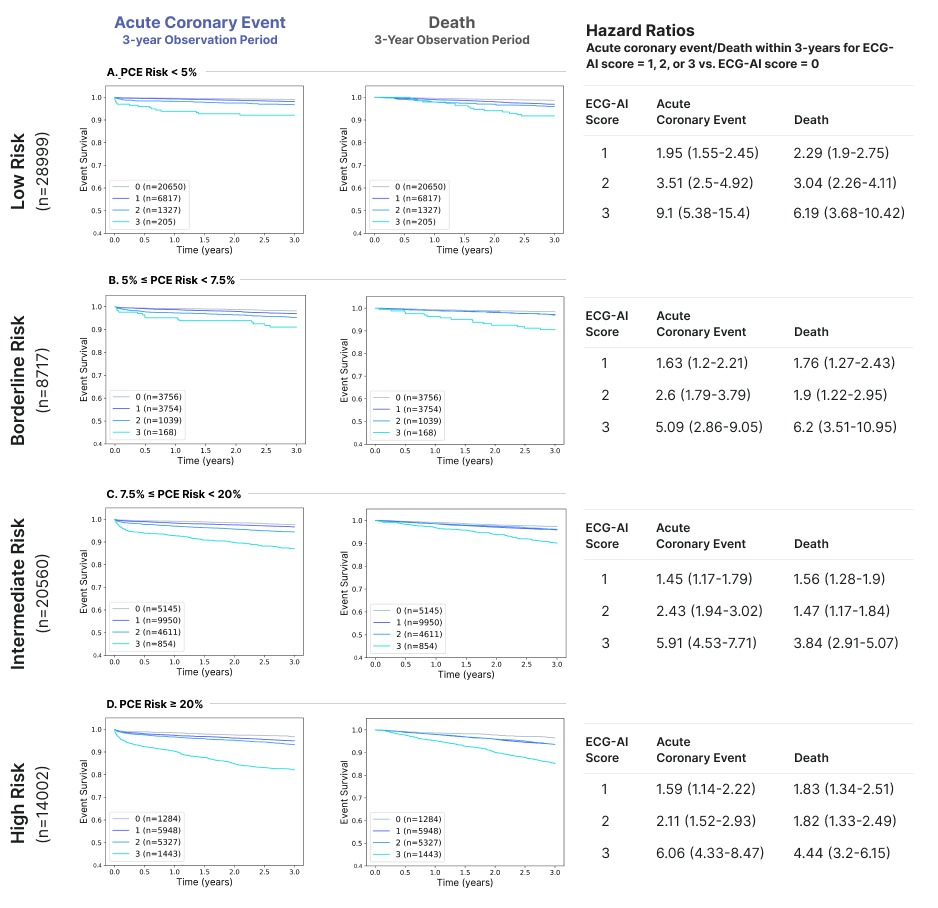


**Supplementary Figure 5.** Event survival curves and hazard ratios for acute coronary events and all-cause death within a 3-year observation period in patients with low (first row), borderline (second row), intermediate (third row), or high (fourth row) ASCVD risk as determined by the PCE. Patients were censored upon loss to follow-up if there was no EHR evidence of survival beyond the observation period or death during the observation period. All patients were required to have evidence of primary care at Mayo Clinic and no history of ASCVD (acute coronary syndrome, peripheral arterial disease, stroke, PCI, CABG, or carotid artery intervention) prior to cohort entry. “ECG-AI score” is defined as the number of positive results on the three disease-specific models.

**Supplementary Figure 6**


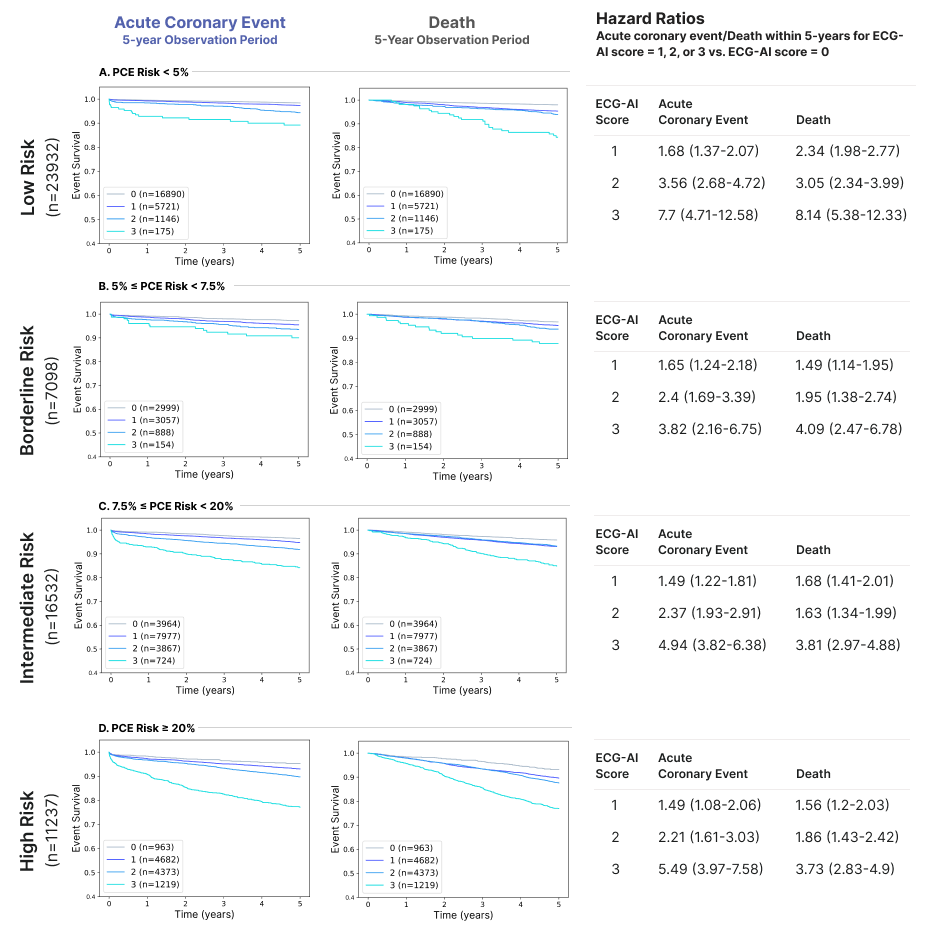
**Supplementary Figure 6.** Event survival curves and hazard ratios for acute coronary events and all-cause death within a 5-year observation period in patients with low (first row), borderline (second row), intermediate (third row), or high (fourth row) ASCVD risk as determined by the PCE. Patients were censored upon loss to follow-up if there was no EHR evidence of survival beyond the observation period or death during the observation period. All patients were required to have evidence of primary care at Mayo Clinic and no history of ASCVD (acute coronary syndrome, peripheral arterial disease, stroke, PCI, CABG, or carotid artery intervention) prior to cohort entry. “ECG-AI score” is defined as the number of positive results on the three disease-specific models.

**Supplementary Figure 7**
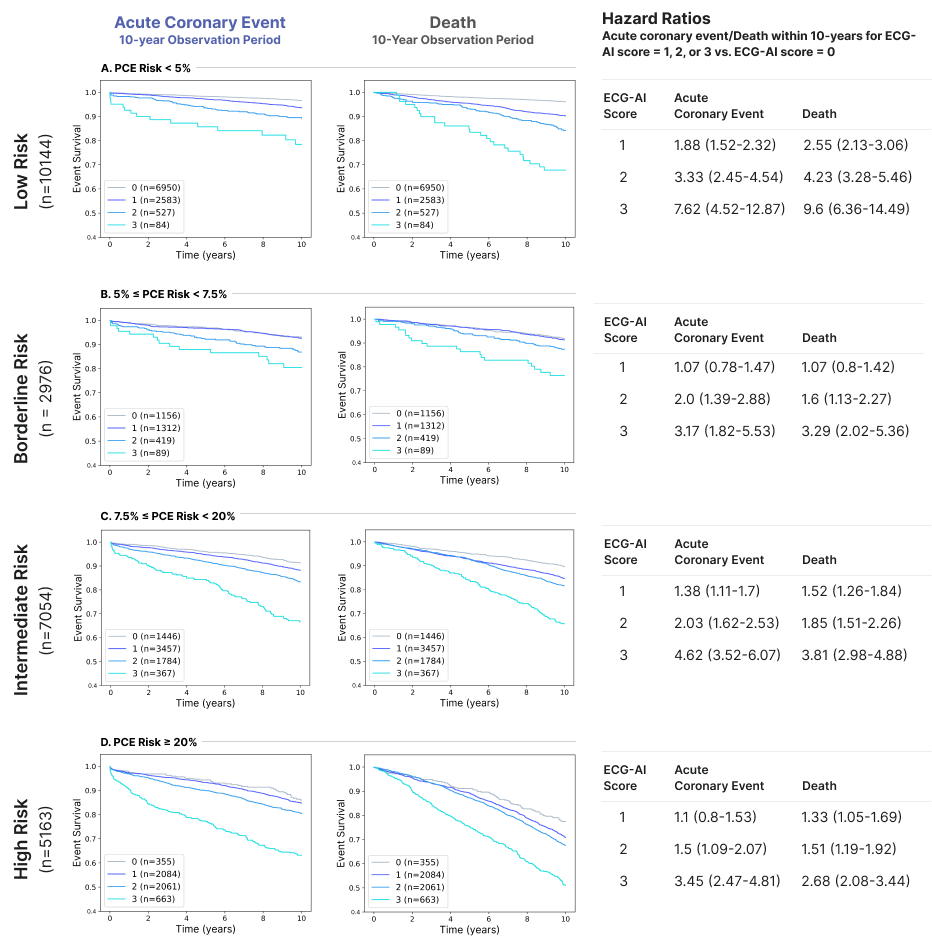


**Supplementary Figure 7.** Event survival curves and hazard ratios for acute coronary events and all-cause death within a 10-year observation period in patients with low (first row), borderline (second row), intermediate (third row), or high (fourth row) ASCVD risk as determined by the PCE. Patients were censored upon loss to follow-up if there was no EHR evidence of survival beyond the observation period or death during the observation period. All patients were required to have evidence of primary care at Mayo Clinic and no history of ASCVD (acute coronary syndrome, peripheral arterial disease, stroke, PCI, CABG, or carotid artery intervention) prior to cohort entry. “ECG-AI score” is defined as the number of positive results on the three disease-specific models.

**Supplementary Figure 8**


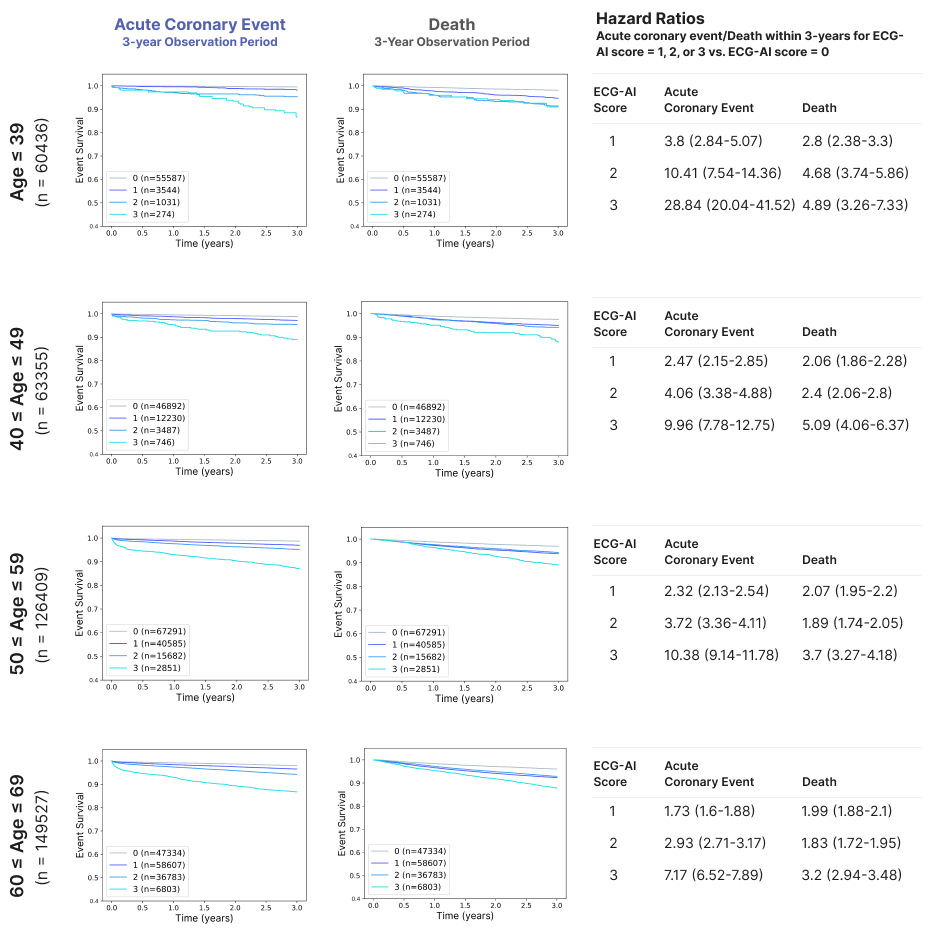


**Supplementary Figure 8.** Event survival curves and hazard ratios for acute coronary events and all-cause death within a 3-year observation period in cohorts stratified by age. “ECG-AI score” is defined as the number of positive results on the three disease-specific models.

**Supplementary Table 3**

|  | **3-year** | **5-year** | **10-year** |  |  | **3-year** | **5-year** | **10-year** |
| --- | --- | --- | --- | --- | --- | --- | --- | --- |
| **Full Cohort Count** | 274479 | 238791 | 120075 |  | **Count with ECG Before ACE*** | 2999 | 4459 | 6642 |
| **Stress Test** |  |  |  |  | **CAC ECG-AI** |  |  |  |
| Count with Stress Test | 25548 | 32073 | 29656 |  | % Pos on CAC | 87.56 | 88.16 | 89.9 |
| % with stress test | 9.31 | 13.43 | 24.7 |  | Mean Time to 1st Pos | 0.603 | 1.096 | 2.216 |
| Mean Time to Stress Test | 1.128 | 1.985 | 3.991 |  | Median Time to 1st Pos | 0.258 | 0.625 | 1.37 |
| Median Time to Stress Test | 1.028 | 1.869 | 3.487 |  | 25 %ile time to 1st Pos | 0 | 0.031 | 0.296 |
| 25 %ile Time to Stress Test | 0.111 | 0.549 | 1.398 |  | 75 %ile time to 1st Pos | 0.994 | 1.797 | 3.484 |
| 75 %ile time to stress test | 1.981 | 3.26 | 6.419 |  | **Obstructive CAD ECG-AI** |  |  |  |
| **CTCA** |  |  |  |  | % Pos on ObCAD | 52.25 | 53.26 | 56.61 |
| Count with CTCA | 1235 | 1517 | 1851 |  | Mean Time to 1st Pos | 0.676 | 1.185 | 2.63 |
| % with CTCA | 0.45 | 0.64 | 1.54 |  | Median Time to 1st Pos | 0.362 | 0.802 | 1.811 |
| Mean Time to CTCA | 1.079 | 2.229 | 6.067 |  | 25 %ile time to 1st Pos | 0.001 | 0.087 | 0.481 |
| Median Time to CTCA | 0.916 | 2.227 | 6.726 |  | 75 %ile time to 1st Pos | 1.139 | 1.921 | 4.343 |
| 25 %ile Time to CTCA | 0.051 | 0.522 | 3.714 |  | **Regional Akinesis ECG-AI** |  |  |  |
| 75 %ile time to CTCA | 1.97 | 3.747 | 8.789 |  | % Pos on Akinesis | 45.25 | 44.97 | 44.53 |
| **ECG** |  |  |  |  | Mean Time to 1st Pos | 0.694 | 1.321 | 3.053 |
| Count with ECG | 99549 | 113850 | 85994 |  | Median Time to 1st Pos | 0.375 | 0.87 | 2.29 |
| % with ECG | 36.27 | 47.68 | 71.62 |  | 25 %ile time to 1st Pos | 0.001 | 0.111 | 0.596 |
| Mean Time to ECG | 0.954 | 1.675 | 3.143 |  | 75 %ile time to 1st Pos | 1.139 | 2.207 | 5.128 |
| Median Time to ECG | 0.718 | 1.299 | 2.316 |  | **0 Pos** |  |  |  |
| 25 %ile Time to ECG | 0.017 | 0.195 | 0.584 |  | % with 0 Pos | 11.2 | 10.79 | 9.41 |
| 75 %ile time to ECG | 1.706 | 2.884 | 5.29 |  | **1 Pos** |  |  |  |
|  |  |  |  |  | % at least 1 Pos | 88.8 | 89.21 | 90.59 |
|  | **3-year** | **5-year** | **10-year** |  | Mean Time to 1 Pos | 0.6 | 1.09 | 2.202 |
| **Cohort Count With ACE*** | 5204 | 7210 | 8936 |  | Median Time to 1 Pos | 0.252 | 0.619 | 1.355 |
| **Stress Test** |  |  |  |  | 25 %ile time to 1 Pos | 0 | 0.027 | 0.29 |
| Count with Stress Test Before ACE | 1158 | 1886 | 3080 |  | 75 %ile time to 1 Pos | 0.992 | 1.783 | 3.458 |
| % with stress test Before ACE | 22.25 | 26.16 | 34.47 |  | **2 Pos** |  |  |  |
| Mean Time to Stress Test (Before ACE) | 0.842 | 1.474 | 3.135 |  | % at least 2 pos | 63.05 | 63.67 | 65.93 |
| Median Time to Stress Test (Before ACE) | 0.558 | 1.102 | 2.455 |  | Mean Time to 2 Pos | 0.671 | 1.209 | 2.646 |
| 25 %ile Time to Stress Test (Before ACE) | 0.037 | 0.154 | 0.933 |  | Median Time to 2 Pos | 0.346 | 0.804 | 1.851 |
| 75 %ile time to stress test (Before ACE) | 1.479 | 2.464 | 5.059 |  | 25 %ile time to 2 Pos | 0.001 | 0.083 | 0.477 |
| **CTCA** |  |  |  |  | 75 %ile time to 2 Pos | 1.115 | 1.984 | 4.344 |
| Count with CTCA Before ACE | 112 | 127 | 221 |  | **3 Pos** |  |  |  |
| % with CTCA Before ACE | 2.15 | 1.76 | 2.47 |  | % 3 pos | 31.81 | 31.49 | 31.09 |
| Mean Time to CTCA (Before ACE) | 0.755 | 1.854 | 5.276 |  | Mean Time to 3 Pos | 0.698 | 1.314 | 3.111 |
| Median Time to CTCA (Before ACE) | 0.258 | 1.17 | 5.847 |  | Median Time to 3 Pos | 0.396 | 0.872 | 2.309 |
| 25 %ile Time to CTCA (Before ACE) | 0.02 | 0.062 | 2.529 |  | 25 %ile time to 3 Pos | 0.002 | 0.115 | 0.62 |
| 75 %ile time to CTCA (Before ACE) | 1.391 | 3.49 | 7.958 |  | 75 %ile time to 3 Pos | 1.158 | 2.129 | 5.317 |
| **ECG** |  |  |  |  | **Conventional ECG** |  |  |  |
| Count with ECG Before ACE | 2999 | 4459 | 6642 |  | % pos by conventional ECG | 13.14 | 14.96 | 17.95 |
| % with ECG Before ACE | 57.63 | 61.84 | 74.33 |  | Mean Time to Conv ECG Pos | 0.899 | 1.669 | 3.731 |
| Mean Time to ECG (Before ACE) | 0.599 | 1.06 | 2.036 |  | Median Time to Conv ECG Pos | 0.697 | 1.227 | 3.305 |
| Median Time to ECG (Before ACE) | 0.258 | 0.589 | 1.199 |  | 25 %ile time to Conv ECG Pos | 0.154 | 0.394 | 1.183 |
| 25 %ile Time to ECG (Before ACE) | 0 | 0.027 | 0.249 |  | 75 %ile time to Conv ECG Pos | 1.475 | 2.755 | 5.895 |

**ACE: Acute Coronary Event*

**Supplementary Table 4**

| **Coronary Artery Calcification** | | | |
| --- | --- | --- | --- |
| Lab | Cohen's D | Risk Increase  Median [IQR] | Risk Decrease  Median [IQR] |
| Creatinine [Mass/volume] in Serum or Plasma | 0.26 | 1.0 [0.8-1.1] | 0.9 [0.7-1.0] |
| Erythrocyte Distribution Width (RDW) [ratio] | 0.2 | 13.6 [13.0-14.7] | 13.4 [12.9-14.3] |
| Platelets [#/volume] in Blood | -0.15 | 219.5 [172.4-271.3] | 231.5 [187.8-279.6] |
| Potassium in serum /plasma | 0.11 | 4.3 [4.0-4.6] | 4.3 [4.0-4.6] |
|  |  |  |  |
| **Obstructive CAD** | | | |
| Lab | Cohen's D | Risk Increase  Median [IQR] | Risk Decrease  Median [IQR] |
| Hematocrit | 0.18 | 40.3 [34.4-43.8] | 38.9 [32.9-42.7] |
| Erythrocyte Distribution Width (RDW) [ratio] | -0.14 | 13.5 [12.9-14.4] | 13.7 [13.0-14.7] |
| Sodium in serum/plasma | -0.11 | 139.4 [137.2-141.3] | 140.0 [137.4-142.0] |
|  |  |  |  |
|  |  |  |  |
| **Regional left ventricular akinesis as possible prior MI** | | | |
| Lab | Cohen's D | Risk Increase  Median [IQR] | Risk Decrease  Median [IQR] |
| Erythrocyte Distribution Width (RDW) [ratio] | 0.31 | 14.0 [13.2-15.4] | 13.6 [13.0-14.5] |
| Hematocrit | -0.17 | 37.8 [31.2-42.5] | 39.3 [33.4-43.0] |
| Creatinine [Mass/volume] in Serum or Plasma | 0.16 | 1.0 [0.8-1.2] | 1.0 [0.8-1.2] |
| Sodium in serum/plasma | -0.15 | 139.3 [137.0-141.3] | 140.0 [137.4-141.5] |
|  |  |  |  |
|  |  |  |  |

**Supplementary Table 4:** Laboratory Correlates of ECG-AI Classification When Discordant/Indeterminate with PCE-based Risk. Shown are the most significant differences in laboratory results within -30 days to +30 days cohort entry in (A) Low or indeterminate risk for ASCVD (< 20%) but ECG-AI positive, versus (B) Indeterminate or high risk for ASCVD (> 7.5%) but ECG-AI negative. Cohen’s D > 0 indicates a higher level in A versus B. Results are limited to labs present in over 50% of the cohorts and with |Cohen’s D| ≥ 0.10.
